# Perspectives of Healthcare Students on Childhood Vaccines: Insights from a Cross-Sectional Study in Bulgaria

**DOI:** 10.3390/vaccines13080804

**Published:** 2025-07-29

**Authors:** Maria Rohova, Nikolay L. Mihaylov, Antoniya Dimova, Rouzha Pancheva

**Affiliations:** 1 Department of Healthcare Economics and Management, Faculty of Public Health, Medical University of Varna, 9002 Varna, Bulgaria; mariarohova@abv.bg (M.R.); n.mihailov@gmail.com (N.L.M.); ant_dimova@abv.bg (A.D.); 2 Department of Hygiene and Epidemiology, Faculty of Public Health, Medical University of Varna, 9002 Varna, Bulgaria

**Keywords:** childhood vaccines, healthcare students, attitudes, misconceptions, Bulgaria

## Abstract

Background/Objectives: Medical and nursing students, as future healthcare professionals, influence public trust and vaccine acceptance. Knowledge gaps or misconceptions regarding immunization may undermine their confidence and effectiveness in addressing vaccine hesitancy. This study explores perceptions and attitudes toward childhood vaccination among Bulgarian healthcare students and factors shaping these outcomes. Methods: A cross-sectional survey was conducted in 2024, using an online self-administered questionnaire completed by 374 medical and nursing students. Descriptive statistics were used to analyze vaccine-related responses, comparing attitudes between healthcare programs and education years. Binomial logistic regression was applied to identify predictors of support for mandatory vaccination, first considering demographic and academic variables, and then adding students’ beliefs and common misconceptions. Results: Medical students showed more positive attitudes toward vaccination than nursing students, with 96.8% of medical students versus 89.4% of nursing students believing vaccines are effective (*p* = 0.005). Students in advanced years demonstrated stronger belief in vaccine effectiveness (*p* = 0.038). Additionally, misbeliefs about the measles vaccine causing autism decreased significantly, with most students in higher years rejecting this misconception (*p* = 0.009). Logistic regression revealed that belief in following the vaccine schedule (OR = 22.71; *p* < 0.001) and confidence in vaccine effectiveness (OR = 10.20; *p* < 0.001) were the strongest predictors of support for mandatory vaccination, with attitudinal factors explaining over half of the variance. Conclusions: Healthcare students’ attitudes about vaccination influence public health outcomes, as their perspectives reflect experience and beliefs. Targeted vaccine education helps address misconceptions and improve vaccination rates.

## 1. Introduction

Healthcare professionals play a significant role in influencing public attitudes toward vaccination and addressing vaccine hesitancy. Patients and parents are more likely to follow recommendations from healthcare providers and often rely on them as trusted sources of vaccine-related information [1,2,3,4]. In a time characterized by widespread misinformation, reliable and authoritative sources promoting vaccine uptake have become particularly crucial. The growing skepticism among the public underscores how important it is that future healthcare professionals are well-informed about the vaccines’ importance, safety, and effectiveness, and also well-equipped with practical communication skills [5,6,7].

While numerous studies consistently report generally positive attitudes among medical and nursing students, a growing body of evidence also highlights insufficient knowledge regarding vaccines [7,8,9,10,11,12]. Positive attitudes suggest a broad acceptance of vaccination as an essential public health intervention; however, a lack of in-depth knowledge could undermine immunization efforts in clinical practice. The knowledge deficiencies encompass various aspects of vaccination. Several studies have highlighted gaps in knowledge regarding specific vaccine schedules [7,10] and contraindications for vaccination [8,13]. There are also documented deficiencies in understanding the characteristics of specific vaccines, such as the influenza vaccine, particularly regarding their effectiveness, the importance of annual administration, and the identification of priority target groups [13]. The lack of understanding of vaccine side effects and safety further compounds this issue, with students frequently overestimating the risk of adverse events [8,14,15]. Such gaps in knowledge and training, alongside personal beliefs, may contribute to hesitancy in recommending vaccines or to insufficient confidence in addressing patient concerns.

International studies reveal a diverse landscape of vaccine-related knowledge and attitudes among healthcare students, with levels of hesitancy varying across different regions and healthcare programs. Recent findings highlight a significant proportion of students displaying hesitancy toward routine childhood vaccinations [16] or skepticism regarding emerging immunization technologies [7]. Misconceptions further fuel vaccine hesitancy, as future healthcare professionals associate vaccines with adverse effects and hold misbeliefs about vaccine safety and immune mechanisms [5,12].

Other studies identify lower hesitancy rates and students demonstrating robust knowledge about immunization. However, misconceptions about vaccine effectiveness and the immunization schedule continue to persist [6,12]. While most healthcare students generally exhibit positive attitudes toward vaccination and higher vaccine adherence, knowledge and acceptance related to vaccines vary by program type [6,17].

These findings highlight a critical gap in healthcare education, as future healthcare professionals may not be sufficiently prepared or willing to address vaccine-related concerns. A growing body of research demonstrates the necessity for enhanced vaccination education within healthcare curricula, particularly training that combines theoretical knowledge with communication skills. Students consistently report feeling underprepared to confront vaccine hesitancy in clinical practice, emphasizing the need to expand the scope of immunization-related content in healthcare programs [7,18,19]. Interventions targeting vaccine knowledge and risk-benefit communication can effectively address these gaps [18,20]. Collectively, these studies underscore the significance of context-specific training in healthcare professions education to foster competency and confidence in future practitioners.

Bulgaria has a longstanding tradition of childhood vaccination, supported by a mandatory immunization system. The national program, administered by general practitioners at no cost to patients, covers vaccines for tuberculosis, hepatitis B, diphtheria, tetanus, pertussis, polio, rubella, measles, mumps, Haemophilus influenzae type b, and pneumococcal infections. Since 2012, there has been a noted decline in vaccination coverage, with rates for most mandatory vaccines falling below the 95% target prior to the COVID-19 pandemic [21]. The pandemic period (2020–2021) resulted in additional coverage deterioration [22]. Contributing factors include low vaccine confidence among parents and negative perceptions within vulnerable groups [21,23].

Healthcare students in Bulgaria enroll in diverse programs, including medicine, dentistry, pharmacy, nursing and midwifery, public health and healthcare management, and allied health professions. The education for medical and nursing students follows unified state requirements, establishing a standardized curriculum framework aligned with EU directives [21]. The Bulgarian medical curriculum incorporates immunization education through mandatory subjects, including microbiology in initial years and immunology, epidemiology, and infectious diseases later. Nursing programs similarly integrate immunization education through compulsory subjects such as microbiology and virology in the early years and infectious diseases and epidemiology in the senior years. Elective courses on vaccines and immunization may be available during education; however, these vary across universities.

Research conducted among healthcare students in Bulgaria regarding COVID-19 vaccines has revealed a concerning prevalence of misconceptions and hesitation. Although students generally display more positive attitudes towards vaccination compared to the general public, they still harbor doubts about vaccine effectiveness and safety [24,25]. Gaining a deeper understanding of the education practices can provide insights into the factors that shape knowledge, attitudes, and beliefs concerning vaccination.

The aim of this study is to investigate the perceptions and attitudes toward childhood vaccination among medical and nursing students in Bulgaria. Specifically, it seeks to identify gaps in understanding and the persistence of misconceptions about vaccines, while also exploring the influence of various factors on these outcomes.

## 2. Materials and Methods

### 2.1. Study Design and Sample

This study employed a cross-sectional survey design to assess the knowledge and attitudes toward childhood vaccination among current healthcare students in Bulgaria. The survey targeted students from three healthcare programs: Medicine (master program), Nursing (bachelor program), and Midwifery (bachelor program). The survey was conducted in all four medical universities in Bulgaria.

Participants were invited to complete an online self-administered questionnaire via email invitations distributed through a national association of medical students, student councils and universities’ official communication channels. Prospective participants were informed about the study’s purpose, procedures, and the voluntary nature of their participation. No incentives were offered for participation. Data were collected between October and November 2024.

The population for the study consisted of all Bulgarian students actively enrolled in either of the three programs in the four universities: approximately 5000 students in Medicine, 2300 in Nursing and 900 in Midwifery. While there is a 50/50 state-mandated gender quota in the Medicine programs, the other two programs are up to 99% female-dominated. A power analysis was conducted using G*Power (v3.1). To detect a medium effect size (w = 0.3) with 80% power and alpha = 0.05 in chi-square group comparisons, based on the very few previous studies of vaccine acceptance among medical students in Bulgaria [24,25], a minimum sample size of 143 per group was required. Our final sample of 374 students (185 medical, 189 nursing/midwifery) exceeded this threshold, ensuring sufficient power for detecting differences in vaccine-related attitudes and beliefs. A total of 477 respondents engaged with the online questionnaire, and 374 (79%) completed the survey, producing the final sample size. A diverse and quite representative sample of student respondents was achieved (see Table 1 in the Results section).

### 2.2. Study Instrument

The data collection tool was a 50-item online self-administered questionnaire designed to capture information regarding participants’ knowledge, attitudes, and beliefs about childhood vaccination, expectations about vaccine-related education, trust in the healthcare system, and other related areas of research interest. The panel of variables used in this study was based on the constructs in the 3C Model of Vaccine Hesitancy [26] and the State of Vaccine Confidence framework [27].

The main constructs of focus included vaccine confidence, perceived effectiveness and safety of childhood vaccines, particularly those included in the compulsory immunization schedule in Bulgaria, perceptions of vaccine-preventable diseases and beliefs about risks associated with vaccines, and attitudes toward mandatory child immunization. Items were adapted from validated instruments aligned with the two models and modified to reflect the Bulgarian vaccination context. The two items on vaccine recommendation to patients (see Table 2) were adapted from Fernández-Prada et al. [28]; the items about misconceptions (vaccines causing autism and vaccines being given too early) and about perceived vaccine benefit/risk balance were adapted from Verger et al. [29]; and all other items were adapted from Larson et al.’s instrument [27]. The questionnaire was administered in Bulgarian and underwent pre-testing for clarity and cultural relevance. Participants responded to statements using a five-point Likert scale, ranging from “Strongly Agree” to “Strongly Disagree”. Data on age, gender, year of study, and program (Medicine, Nursing, or Midwifery) were also collected.

### 2.3. Statistical Analyses

For the purposes of the analysis, healthcare students were stratified into two groups: the first including medical students and the second students in Nursing and Midwifery (labeled “nursing students” in the presentation of the analyses). The five-point scale measuring vaccine acceptance and confidence was dichotomized to distinguish confident vs. hesitant attitudes toward vaccines. Answers “Completely agree” and “Rather agree” were merged into “Agree”, answers “Completely disagree” and “Rather disagree” were merged into “Disagree” and the “Not sure” answer was merged into one of the remaining categories depending on whether it means confidence or hesitance toward vaccines depending on the item formulation.

Descriptive statistics were employed to summarize the demographic characteristics of the participants and their responses to vaccine-related items. Differences in knowledge and attitudes toward childhood vaccination between the healthcare programs and years of education were explored through the Pearson chi-square test for categorical variables. Effect sizes were estimated using the Phi coefficient for categorical comparisons.

A two-step binomial logistic regression analysis was performed to identify predictors of support for mandatory vaccination (*I would recommend mandatory vaccines on the immunization schedule in my future practice*). The dependent variable (support for mandatory vaccination) was transformed into a binary outcome (Yes/No). Predictors were added hierarchically across models: Model 1 included relevant non-modifiable demographic and contextual academic characteristics (age, program, year of study), while Model 2 added core vaccine attitudes such as confidence in the vaccines’ effectiveness and safety, beliefs about vaccines’ importance [27], as well as misconceptions about vaccines. Model performance was assessed through McFadden’s R^2^, Akaike Information Criterion (AIC) and Bayesian Information Criterion (BIC). Odds ratios (OR) and 95% confidence intervals (CI) were estimated for each predictor. Statistical significance was set at *p* < 0.05. Data analysis was performed using *jamovi* (ver. 2.4).

## 3. Results

### 3.1. Demographic Characteristics

Among the 374 students participating in the survey, there were 185 students in Medicine (49.5%), 157 in Nursing (42%), and 32 in Midwifery (8.6%). Female respondents predominated in the nursing (96.2%) and midwifery (96.9%) groups, compared to 64.9% in the medical group. The average age differed significantly across groups, with nursing students being older (M = 30.0, SD = 11.25) than medical (M = 23.1, SD = 5.65) and midwifery students (M = 25.3, SD = 7.47). Most medical students were in their third year or above (69.7%), in contrast to nursing students, 82.8% of whom were in their first or second year. All three programs (Medicine, Nursing, Midwifery) are statistically different on each baseline characteristic, *p* < 0.001.

**Table 1:** Demographic characteristics of student respondents (N = 374).

	Medicine n	%	Nursing n	%	Midwifery n	%	Total n	%
**Gender**								
Female	120	64.9	151	96.2	31	96.9	302	80.7
Male	63	35.1	4	3.8	1	3.1	68	18.2
**Age group**								
Under 20 years	23	12.4	30	19.1	2	6.3	55	14.7
20–25 years	145	78.4	49	31.2	21	65.6	215	57.5
Above 25	17	9.2	78	49.7	9	28.1	104	27.8
**Year of Study**								
1st and 2nd	56	30.3	130	82.8	14	43.8	200	53.5
3rd and above	129	69.7	27	17.2	18	56.3	174	46.5

Note: Missing data—Gender: 4 (1.1%), Age group: 0 (0.0%), Year of study: 0 (0.0%).

### 3.2. Group Differences in Vaccine Attitudes

Medical students expressed consistently more favorable attitudes toward vaccination compared to nursing students based on their stance on immunization-related statements (Table 2). Agreement with the statement that vaccines are generally effective (belief in vaccine effectiveness) was nearly universal among medical students (96.8%), while somewhat lower among nursing students (89.4%; *p* = 0.005, Phi = 0.144). Nursing students were significantly more likely to have misconceptions, namely to believe that the measles vaccine can cause autism (65.6% vs. 40.5%) and to believe that children are vaccinated too early for their age (47.6% vs. 14.1%), and also to question the dangers of vaccine-preventable diseases (disease complacency), the benefits of vaccines vs. the potential risks (confidence), and the importance of the immunization schedule (all *p* < 0.001; Phi ranging from 0.207 to 0.363). Additionally, medical students showed stronger support for mandatory vaccination (95.1% vs. 85.7%; *p* = 0.002), whereas support for voluntary recommended vaccination was more common among nursing students (*p* = 0.003).

**Table 2:** Group differences in vaccine beliefs and attitudes.

	Medicine Students		Nursing Students		*p*-Value ^1^	Phi ^2^
	n	%	n	%		
**Overall, vaccines are effective for disease prevention**						
Agree	179	96.8	169	89.4		
Disagree/Not sure	6	3.2	20	10.6	0.005	0.144
**The measles vaccine can cause autism in children**						
Disagree	110	59.5	65	34.4		
Agree/Not sure	75	40.5	124	65.6	<0.001	0.251
**Children are vaccinated too early for their age**						
Disagree	159	85.9	99	52.4		
Agree/Not sure	26	14.1	90	47.6	<0.001	0.363
**Everyone should be able to choose whether to vaccinate or not**						
Disagree	100	54.1	55	29.1		
Agree/Not sure	85	45.9	134	70.9	<0.001	0.253
**Most vaccine-preventable diseases are dangerous**						
Agree	152	82.2	116	61.4		
Disagree/Not sure	33	17.8	73	38.6	<0.001	0.231
**The benefits of vaccines outweigh the potential risks**						
Agree	133	71.9	77	40.7		
Disagree/Not sure	52	28.1	112	59.3	<0.001	0.314
**Full protection can be achieved only if the regular mandatory schedule is followed**						
Agree	157	84.9	127	67.2		
Disagree/Not sure	28	15.1	62	32.8	<0.001	0.207
**I would recommend to my patients to comply with the mandatory schedule**						
Agree	176	95.1	162	85.7		
Disagree/Not sure	9	4.9	27	14.3	0.002	0.160
**I would advise my patients to take the recommended vaccinations**						
Agree	156	84.3	135	71.4		
Disagree/Not sure	29	15.7	54	28.6	0.003	0.155

^1^ Pearson chi-square test for categorical variables, *p* ≤ 0.05. ^2^ Values of Phi: 0.1–0.3—small, 0.3–0.5—medium, above 0.5—large effect size [30].

### 3.3. Education Year Differences in Vaccine Attitudes

Students further ahead in their healthcare education expressed consistently more favorable attitudes toward vaccination compared to first- and second-year students (Table 3). Agreement with the statement that vaccines are generally effective (belief in vaccine effectiveness) was high in lower-year students and even higher in more educated students (*p* = 0.038, Phi = 0.107). More substantial changes in attitudes seem to transpire along misconception beliefs about the measles vaccine and autism, where the majority balance shifts (*p* = 0.009, Phi = 0.135); the confidence in the safety of the vaccines (*Children are vaccinated too early for their age*), with an effect size of 0.231; the conviction in the importance of vaccines and their mandatory nature (Phi = 0.173); and the perceived positive balance of benefits and risks of vaccines, where the balance shifts as well (Phi = 0.176). The analysis of other vaccine-related statements also conveys more positive attitudes among higher-level students, the only non-significant statement being about the dangers of preventable diseases—here the attitudes are positive as well, but the difference is just below the significance threshold (*p* = 0.056).

### 3.4. Predictors of Supportive Attitudes to Mandatory Vaccination

A hierarchical logistic regression analysis was conducted to identify factors associated with support for mandatory childhood vaccination. Results from the logistic regression analysis are summarized in Table 4. Model 1, which included non-modifiable demographic and contextual covariates, showed a modest model performance and low explanatory power (R^2^ = 0.052, *p* = 0.015). Adding core beliefs in Model 2 substantially improved model performance and increased tenfold the explanatory power (R^2^ = 0.518, *p* < 0.001), with the values of both AIC and BIC decreasing substantially.

In Model 1, being a medical student was associated with higher odds of supporting mandatory vaccination compared to nursing students (OR = 0.32, 95% CI: 0.13–0.83, *p* = 0.019), indicating that nursing students were less likely to endorse this policy and thus can be considered to have somewhat less positive attitudes toward vaccines. Other demographic and contextual covariates, age group and level of education, were not associated with vaccine attitudes.

In Model 2, attitudinal variables were added, providing a tenfold increase in the R-squared value and explaining 51.8% of the variance. The effect of program was no longer significant, suggesting that differences in support for mandatory vaccination were better explained by attitudinal rather than contextual factors.

As would be expected, respondents who believed that full protection from disease comes only with full compliance with the vaccine schedule also had over 22 times greater odds of supporting mandatory childhood vaccination (OR = 22.71, 95% CI: 6.55–78.72, *p* < 0.001); this attitude was the strongest predictor. Another strong and significant predictor emerged to be the confidence in the vaccines’ effectiveness. Participants who agreed with that statement were over ten times more likely to support mandatory vaccination (OR = 10.20, 95% CI: 3.86–26.97, *p* < 0.001).

Statements tapping into beliefs about the vaccines’ importance were also positively associated with the general support for vaccines. Disagreeing with the idea that individuals should choose whether to take up mandatory vaccines (OR = 7.27, 95% CI: 1.41–37.54, *p* = 0.018) and agreeing with the belief that vaccine-preventable diseases are dangerous (OR = 5.59, 95% CI: 1.82–17.13, *p* = 0.003) were both associated with increased support for mandatory vaccination. Confidence in the vaccines’ safety, expressed in the disagreement with the statement that children are vaccinated too early, was linked to significantly higher odds of support (OR = 6.37, 95% CI: 2.43–16.71, *p* < 0.001).

Two attitude-related predictors emerged as non-significant in Model 2. The belief that the measles vaccine causes autism (a misconception attitude) was not a significant predictor (*p* = 0.206). The vaccine confidence statement that the benefits outweigh the risks of vaccines, which is a composite belief in the vaccines’ effectiveness and safety, had no unique contribution to the outcome of the regression. Model 2 highlights the primary role of belief-based factors in shaping students’ attitudes toward mandatory vaccination.

## 4. Discussion

In the present study, we examined the perceptions and attitudes of medical and nursing students towards childhood vaccination. Our findings revealed generally positive attitudes among healthcare students toward vaccines, with the majority of them supporting vaccination and acknowledging its importance. Most participants recognized vaccines as effective: nearly all medical students (96.8%) agreed that vaccines are generally effective, compared to 89.4% of nursing students. There is broad support for the immunization schedule, although some students preferred voluntary vaccination over mandates. The vast majority would also advise parents to follow the immunization schedule.

Medical students and those further in their healthcare education displayed stronger pro-vaccination attitudes, fewer misconceptions, and greater support for mandatory immunization policies compared to nursing students and those earlier in training. Medical students consistently held favorable attitudes toward vaccination, showing stronger belief in vaccine effectiveness and stronger support for mandatory vaccination, while nursing students were more likely to favor voluntary vaccination.

Previous research consistently demonstrates that medical students generally exhibit positive attitudes and supportive practices toward vaccination [11,31], with a marked increase in these attitudes observed during the final year of study [18]. Across healthcare disciplines, most students report favorable views regarding the importance and necessity of immunization, and recognize the effectiveness of vaccines [7,14,31,32]. The majority express willingness to recommend childhood vaccination to parents [32]. While students in nursing and other healthcare programs also tend to hold positive attitudes toward vaccination [33], the proportion expressing strong support is somewhat lower compared to medical students.

While overall attitudes toward vaccination among healthcare students were positive, a notable minority remained hesitant, often due to safety concerns. The concerns surrounding vaccine side effects and safety became more pronounced when discussing specific vaccines. A considerable proportion of students held the misconception that the measles vaccine is linked to autism. Nursing students were significantly more likely to agree that the measles vaccine can cause autism (65.6% of nursing students vs. 40.5% of medical students) and that children are vaccinated too early (47.6% vs. 14.1%).

Various international studies have identified misbeliefs in the association between vaccines and chronic diseases or severe side reactions. Some healthcare students maintain beliefs linking vaccines to chronic diseases [8,16,34] and severe adverse effects [7], or believe that vaccines are unnecessary due to the superiority of natural immunity [12]. A study conducted among nursing and midwifery students in Italy found that a significant proportion hold misconceptions regarding vaccine safety, including the belief that influenza vaccines could cause the illness or that adjuvants might trigger severe side effects [5]. The widespread dissemination of misinformation, particularly through social media, has been shown to influence attitudes and perceptions among younger populations [35]. Exposure to inaccurate claims may exacerbate hesitancy, as students often lack the confidence or knowledge to counter misinformation effectively.

The current study identified differences between academic programs. Nursing students generally held positive attitudes toward vaccination, but these attitudes were not as strong or consistent as those of medical students. The divergence in opinions regarding the severity of disease risks highlighted the multitude of possibilities of students’ attitudes. Nursing students assessed the risks as lower, which influenced the perceived necessity and importance of vaccination. This underestimation of disease severity may lead to a reduced appreciation of preventive measures, including immunization. Furthermore, such perceptions appear to influence the perceived risk-benefit balance of vaccines, resulting in some of the surveyed students being unable to assume a definitive stance in favor of vaccination.

Evidence indicates divergent attitudes between medical and nursing students, though the extent and patterns of these differences vary across studies and vaccine types. For instance, Dybsand et al. have found that final-year nursing students demonstrate suboptimal performance across several topics related to immunization and harbor reservations about the vaccine risk-to-benefit ratio [6]. Similarly, medical and pharmacy students in Canada have displayed more pro-vaccination attitudes and higher vaccine acceptance than nursing students [17]. A study examining Romanian healthcare students’ attitudes toward COVID-19 vaccination has revealed that medical students were more likely to support vaccination compared to their peers in dentistry, pharmacy, and nursing [32]. Differences in COVID-19 vaccine acceptance have been found in a study conducted in Bulgaria, with medical students showing a greater willingness to be vaccinated than nursing or rehabilitation students [24]. Additionally, medical students generally demonstrate higher knowledge and more positive attitudes toward child immunization, though areas requiring improvement still remain [7,8,9].

Given the consistent findings across multiple studies, hesitancy is more common among students with lower levels of knowledge, usually those in earlier years of study. Knowledge serves as a significant predictor of vaccine-related attitudes, with evidence indicating a positive correlation between greater knowledge and more favorable perceptions of vaccination [8,34,36]. Our findings revealed that students in advanced years of study tended to have stronger trust in vaccines and were more likely to acknowledge the benefits of immunization. Students further along in their studies demonstrated increased belief in vaccine effectiveness, reduced misconceptions (especially regarding the measles vaccine and autism), and a stronger conviction in the importance and mandatory nature of vaccines. Furthermore, more advanced students showed a more positive assessment of the balance between vaccine benefits and risks than those in their first or second years.

This pattern is further supported by research indicating that vaccine hesitancy among medical students has declined as they progress through their academic training [11,37], with higher years of study consistently associated with increased vaccine acceptance [38]. The trend suggests that enhanced knowledge may contribute to a more informed perspective on vaccination. Additionally, as students advance in their studies, they may develop stronger critical thinking skills, enabling them to distinguish between scientific evidence and misinformation regarding vaccines.

According to our findings, support for mandatory vaccination was primarily determined by attitudinal rather than demographic or educational variables. Medical students were more likely than nursing students to support mandatory vaccination, but age and education level showed no significant association. After accounting for attitudes, the difference between medical and nursing students was no longer significant, highlighting that beliefs primarily drive support. Personal beliefs and perceived risk of vaccine-preventable diseases influenced vaccine attitudes, with trust in the mandatory immunization schedule and confidence in vaccine effectiveness as strong predictors of promoting vaccination benefits in future practice. Furthermore, acceptance was associated with belief in scientific evidence supporting vaccine safety and effectiveness, as well as confidence in healthcare authorities and national immunization programs [32]. Consistent with the existing literature, greater knowledge about vaccines, particularly regarding their safety profiles and effectiveness, correlated with higher acceptance rates [7,8,18].

Studies consistently emphasize that targeted educational interventions can effectively enhance vaccine acceptance and confidence among healthcare students [14,18,39]. These interventions often focus on improving communication skills and correcting common misconceptions. Curricular reforms in medical and nursing education should integrate scientific instruction on vaccine mechanisms into practical training in vaccine promotion and countering misinformation. Better understanding of students’ attitudes toward vaccination could inform the development of training programs to improve the preparedness of future healthcare professionals in tackling immunization-related challenges.

While this study provides valuable insights into Bulgarian healthcare students’ attitudes toward childhood vaccination, there are several limitations to consider. The cross-sectional design precludes causal inference regarding observed associations between variables. The sample size and demographic composition of the participants may not be fully representative of all Bulgarian healthcare students, potentially limiting the generalizability of the findings. Furthermore, the study did not account for cultural factors relevant to the Bulgarian context that could shape vaccination attitudes. Future research could benefit from longitudinal studies that follow students over time to observe changes in attitudes and knowledge as they progress through their education.

## 5. Conclusions

Our study’s findings indicate predominantly positive attitudes among Bulgarian medical and nursing students regarding the effectiveness and necessity of childhood vaccinations. While both groups exhibited pro-vaccination attitudes, medical students demonstrated notably stronger support for mandatory immunization programs and were less prone to misconceptions about potential side effects. The study also revealed an association between educational progression and more favorable attitudes towards vaccination, with students in advanced years of study showing greater confidence in vaccine safety. These attitudes are shaped by a complex interplay of factors, including individual beliefs and pre-existing perceptions about immunization.

The attitudes of healthcare students towards childhood vaccination are of significant importance, as they are future influencers of public health decisions. Identifying specific misconceptions among healthcare students highlights the need for targeted educational initiatives that focus on childhood vaccination, thereby enhancing future healthcare professionals’ vaccination confidence.

## Figures and Tables

**Table 3:** Education level differences in vaccine attitudes.

	1st and 2nd Year		3rd Year and Above		*p*-Value ^1^	Phi ^2^
	n	%	n	%		
**Overall, vaccines are effective for disease prevention**						
Agree	181	90.5	167	96.0		
Disagree/Not sure	19	9.5	7	4.0	0.038	0.107
**The measles vaccine can cause autism in children**						
Disagree	81	40.5	94	54.0		
Agree/Not sure	119	59.5	80	46.0	0.009	0.135
**Children are vaccinated too early for their age**						
Disagree	118	59.0	140	80.5		
Agree/Not sure	82	41.0	34	19.5	<0.001	0.231
**Everyone should be able to choose whether to vaccinate or not**						
Disagree	67	33.5	88	50.6		
Agree/Not sure	133	66.5	86	49.4	<0.001	0.173
**Most vaccine-preventable diseases are dangerous**						
Agree	135	67.5	133	76.4		
Disagree/Not sure	65	32.5	41	23.6	0.056	-
**The benefits of vaccines outweigh the potential risks**						
Agree	96	48.0	114	65.5		
Disagree/Not sure	104	52.0	60	34.5	<0.001	0.176
**Full protection can be achieved only if the regular mandatory schedule is followed**						
Agree	139	69.5	145	83.3		
Disagree/Not sure	61	30.5	29	16.7	0.002	0.161
**I would recommend to my patients to comply with the mandatory schedule**						
Agree	174	87.0	164	94.3		
Disagree/Not sure	26	13.0	10	5.7	0.018	0.123
**I would advise my patients to take the recommended vaccinations**						
Agree	147	73.5	144	82.8		
Disagree/Not sure	53	26.5	30	11.2	0.032	0.111

^1^ Pearson chi-square test for categorical variables, *p* ≤ 0.05. ^2^ Values of Phi: 0.1–0.3—small, 0.3–0.5—medium, above 0.5—large effect size [30].

**Table 4:** Summary of logistic regression analysis for variables predicting support for mandatory vaccination.

	Model 1		Model 2	
Predictor	OR	*p*-Value	OR	*p*-Value
Age (20–25 y vs. >25 y)	1.14	0.759	0.95	0.927
Age (<20 y vs. >25 y)	0.45	0.236	1.69	0.518
Program (Medicine vs. Nursing)	**0.32**	**0.019**	0.96	0.949
Education Year (≥3rd year vs. <3rd year)	0.76	0.600	1.86	0.455
Vaccines are overall effective (Agree vs. Disagree)			**9.73**	**<0.001**
Benefits outweigh risks (Agree vs. Disagree)			0.60	0.394
Everyone be able to choose whether to vaccinate (Disagree vs. Agree)			**7.27**	**0.018**
Vaccine-preventable diseases are dangerous (Agree vs. Disagree)			**5.59**	**0.003**
Full protection if vaccine schedule followed (Agree vs. Disagree)			**22.71**	**<0.001**
The measles vaccine can cause autism (Disagree vs. Agree)			0.76	0.681
Children are vaccinated too early (Disagree vs. Agree)			**6.56**	**<0.001**
R^2^	0.052		0.518	
AIC	235		138	
BIC	254		185	

Significant values (*p* < 0.05) are shown in bold.

## Data Availability

The data that support the findings of this study are available from the corresponding author upon reasonable request.

## Abbreviations

The following abbreviations are used in this manuscript:

SD	Standard deviation
AIC	Akaike Information Criterion
BIC	Bayesian Information Criterion
OR	Odds ratio
CI	Confidence interval
